# Metabolic Consequences of Advanced Chronic Heart Failure and its Modification by Implantation of a Durable Left Ventricular Assist Device

**DOI:** 10.31083/j.rcm2511388

**Published:** 2024-10-31

**Authors:** Daniel Hlaváček, Martin Haluzík, Jakub Mahrík, Ganna Popivnyak, Barbora J. Kasperová, Peter Ivák

**Affiliations:** ^1^Department of Cardiovascular Surgery, Institute for Clinical and Experimental Medicine, 140 21 Prague, Czech Republic; ^2^Department of Physiology, Third Faculty of Medicine, Charles University, 100 00 Prague, Czech Republic; ^3^Department of Diabetes, Institute for Clinical and Experimental Medicine, 140 21 Prague, Czech Republic; ^4^First Faculty of Medicine, Institute of Medical Biochemistry and Laboratory Diagnostics, Charles University, 128 08 Prague, Czech Republic; ^5^Department of Anesthesia and Resuscitation, Institute for Clinical and Experimental Medicine, 140 21 Prague, Czech Republic; ^6^First Faculty of Medicine, Charles University in Prague, 121 08 Prague, Czech Republic; ^7^Second Department of Surgery, Department of Cardiovascular Surgery, First Faculty of Medicine, Charles University, 150 06 Prague, Czech Republic

**Keywords:** heart failure, mechanical circulatory support, left ventricular assist device, metabolism, obesity, cachexia, gliflozins, diabetes

## Abstract

Heart failure (HF) is a clinical syndrome characterized by the inability of the heart to provide adequate perfusion to tissues and organs, resulting in typical symptoms such as fatigue, dyspnea, dyspepsia, or swelling due to decreased cardiac output. With its increasing prevalence, heart failure has become one of the leading causes of morbidity and mortality worldwide, imposing a significant burden on the population by reducing long-term life expectancy and raising hospital costs. Indeed, over 20 million people worldwide suffer from heart failure, with a 5-year mortality rate of 60–70%. As heart failure progresses, various structural and metabolic changes occur within the myocardium and organ systems. In the past two decades, therapeutic options for heart failure patients have significantly expanded. In addition to novel pharmacological treatment, advanced surgical methods such as heart transplantation (HTx) and the implantation of durable left ventricular assist devices (LVADs) are available for patients with end-stage heart failure. This review discusses the pathophysiological aspects and metabolic consequences of heart failure and metabolic changes, as well as the benefits and challenges of implanting a left ventricular assist device. Furthermore, future targets for heart failure diagnostics and therapy will be highlighted.

## 1. Introduction

Heart failure (HF) is a chronic pathologic state that results from structural or functional abnormalities of the heart, including coronary artery disease, valvular diseases, cardiomyopathies, and arrhythmias. Structural myocardial changes, such as myocardial hypertrophy, are mainly caused by mechanical or volume overload of the heart. Hypertrophy of cardiomyocytes increases oxygen consumption, exacerbating ischemia and deteriorating heart function. These processes may also lead to apoptosis, necrosis, changes in the extracellular matrix, and ventricular remodeling [1].

Chronic heart failure is classified based on left ventricular ejection fraction (LVEF): heart failure with reduced EF (HFrEF, EF <40%); HF with preserved EF (HFpEF, EF >50%); HF with mildly reduced EF (HFmrEF, EF 41–49%) [2].

The primary goal of heart failure therapy is causal. Initially, patients are managed with non-surgical modalities, including lifestyle changes, guideline-directed medical therapy (GDMT), catheter-based therapies (such as percutaneous coronary intervention or radiofrequency ablation), and resynchronization therapy [1]. Suitable patients may undergo standard surgical treatments such as myocardial revascularization or valve procedures [1, 3].

For patients with end-stage heart failure, characterized by progressive severe symptoms, recurrent decompensations, and severe cardiac dysfunction despite medical optimization, advanced surgical therapies are available. The gold standard treatment is heart transplantation [4]; however, the implantation of durable left ventricular assist devices (LVADs) has become the standard of care due to the scarcity of donor hearts. LVADs assist a failing heart in pumping blood or can even replace its function entirely. Additionally, major improvements in device durability and complications profile allow prolonged use of these devices. Therefore, LVAD implantation may be indicated not only as a bridge to transplantation (BTT), bridge to candidacy (BTC), bridge to decision (BTD), and bridge to recovery (BTR) but also as a permanent solution for several patients as a destination therapy (DT) [5].

This review discusses the pathophysiological aspects and metabolic consequences of heart failure and also aims to shed light on metabolic changes, benefits, and pitfalls that may occur after LVAD implantation. Additionally, it focuses on current and future targets for heart failure diagnostics and therapy.

## 2. Pathophysiology and Metabolic Changes in Chronic Heart Failure 

**Fig. 1.**
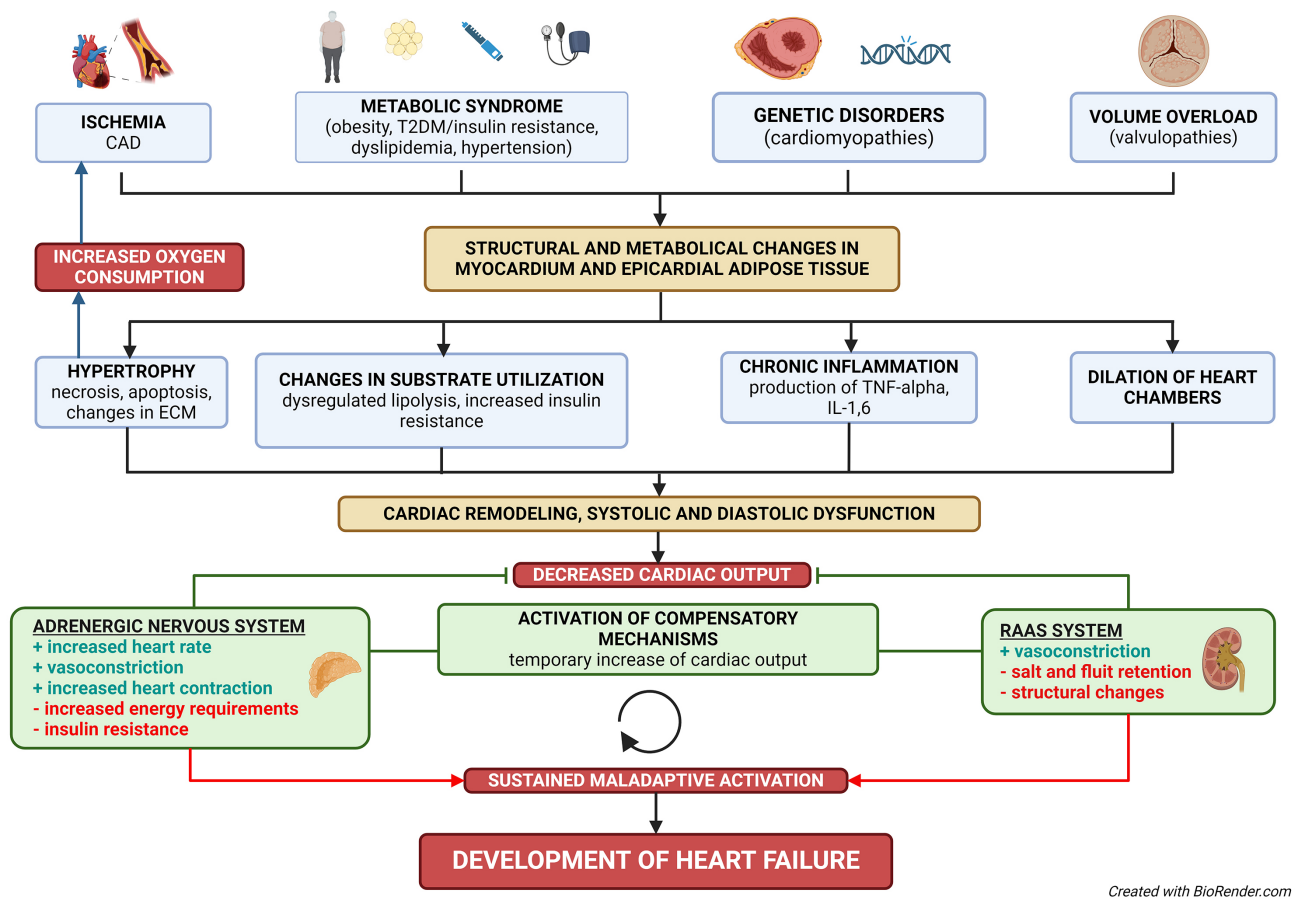
**Structural and metabolic pathways in chronic heart failure**. The diagram illustrates the structural and metabolic pathways leading to chronic heart failure. Key contributing factors include ischemia, metabolic syndrome, genetic disorders (cardiomyopathies), and volume or pressure overload (valvulopathies). These factors cause changes in the myocardium and epicardial adipose tissue, leading to hypertrophy, altered substrate utilization, chronic inflammation, and dilation of heart chambers. The resulting cardiac remodeling causes systolic and diastolic dysfunction, triggering compensatory mechanisms (adrenergic nervous system, RAAS system). Initially, these mechanisms temporarily improve cardiac output, but their sustained activation eventually leads to maladaptive changes and the onset of heart failure. CAD, coronary artery disease; ECM, extracellular matrix; IL-1,6, interleukin 1,6; RAAS system, renin–angiotensin–aldosterone system; T2DM, type 2 diabetes mellitus; TNF-alpha, tumor necrosis factor-alpha.

Decreased cardiac output (CO) activates physiological compensatory mechanisms, such as the adrenergic nervous system or renin–angiotensin–aldosterone system (RAAS), to maintain normal heart function. Sustained activation of compensatory mechanisms is maladaptive and may exacerbate cardiac decompensation [1] (Fig. 1). Activation of the adrenergic nervous system during the early stages of heart failure increases the heart rate, facilitates heart muscle contraction and peripheral vasoconstriction, and augments cardiac output. Sympathetic overdrive further accelerates peripheral vascular resistance and contributes to the development of arterial hypertension. Moreover, myocardial energy requirements increase and could lead to increased levels of circulating norepinephrine and many metabolic changes, such as activation of lipolysis, gluconeogenesis, and increased insulin resistance [6, 7].

In a healthy individual, the heart derives two-thirds of its energy from the oxidation of fatty acids (FAs) in the mitochondria. On the other hand, in the course of chronic heart failure, prolonged activation of compensatory mechanisms leads to a hemodynamic and neurohormonal imbalance. Sustained and dysregulated lipolysis with decreased FA oxidation causes the accumulation of toxic lipid intermediates (diacylglycerols, ceramides) and contributes to insulin resistance in the myocardium. In combination with systemic insulin resistance, there is an essential metabolic shift towards glycolysis as the primary energy source in patients with HF. Throughout all of this, the loss of energy substrates, depletion in adenine nucleotide pool (adenosine mono-, di-, triphosphate – AMP, ADP, ATP), creatine kinase (CK), creatine phosphate (CrP) + CrP/ATP ratio, contribute to the starvation of the failing heart and increased oxidative stress [6, 7, 8]. Several derangements in mitochondrial structure and function were also described, leading to increased production of reactive oxygen species, altered generation of ATP, and cellular death [9].

Renal hypoperfusion leads to RAAS activation, leading to increased sympathetic stimulation of kidneys to maintain circulatory homeostasis via regulation of vasoconstriction and natriuresis. Prolonged RAAS activation leads to the retention of salt and fluids and the development of volume and pressure overload [4]. This promotes a release of counteracting hormones (natriuretic peptides), which causes vasodilation and diuresis. The levels of natriuretic peptides have diagnostic and prognostic importance [10]. Neurohormonal products (angiotensin II, aldosterone) in combination with cytokines, notably tumor necrosis factor-alpha (TNF-α), interleukin-1, and 6 (IL-1,6), play substantial roles in cardiomyocyte apoptosis and myocardial fibrosis. Myocardial injury further causes transcriptional and post-transcriptional changes, including activation of fetal genes and the switch from alfa to beta myosin heavy chain subtypes [7]. All of the above aggravates structural dysfunction of the heart.

Another potential mechanism contributing to the progression of heart failure could be identified in the epicardial adipose tissue (EAT). Changes in EAT metabolic processes were described as involved in the pathophysiology of various cardiovascular diseases, such as coronary artery disease or atrial fibrillation [11, 12, 13]. Epicardial adipose tissue is a highly metabolically active organ located on the surface of the myocardium. It has several vital functions in the heart, e.g., lipid and glucose metabolism regulation, energy storage, protection from unfavorable metabolic (hypoxia, ischemia), and mechanical conditions. Under metabolic stress, environmental conditions, and aging, a harmful imbalance between protective (adiponectin) and damaging (i.e., TNF-α, IL-1, IL-6, IL-8, IL-10) mediators produced in epicardial adipose tissue may occur [11, 12, 13]. These pathological changes augment autocrine and paracrine secretion of proinflammatory factors, leading to increased inflammation, fibrosis, and autonomic dysregulation, which, among others, contributes to systemic insulin resistance. Moreover, the mechanical effect of an enlarged fat layer pad worsens diastolic relaxation and filling [13, 14].

It has been described that epicardial adipose fat tissue volume is increased in obese patients with type 2 diabetes mellitus and coronary artery disease. Therefore, measuring EAT volumes (by ultrasound, computer tomography scan, or magnetic resonance imaging) may serve as a predictor of cardiovascular events [12]. Further, defining a standard measurement method could be a breakthrough in cardiovascular risk evaluation.

Epicardial adipose tissue represents a potentially modifiable cardiovascular risk factor and may be a therapeutical target in heart failure therapy. Novel therapeutic agents such as glucagon-like peptide one receptor (GLP1R) inhibitors of sodium-glucose co-transporter 2 (SGLT2i) may reduce inflammation, increase free fatty acids oxidation, and improve myocardial insulin sensitivity. SGLT2i may induce sympatholytic and lipolytic effects, reducing oxygen consumption in heart failure patients. Dapagliflozin and GLP1R agonists may also reduce epicardial fat layer thickness, improving systolic and diastolic function [11, 13]. However, the pleiotropic beneficial effects of these drugs must be studied further.

### 2.1 Obesity and Heart Failure

Obesity is associated with an increase in both cardiovascular and non-cardiovascular mortality. Mortality from cardiovascular diseases in the obese population is increased about five-fold compared to non-obese individuals. Further, obese patients are more prone to suffer from HFpEF than HFrEF. Overall, heart failure is more likely to occur in women [15]. Additionally, obese patients also wait longer for heart transplants, and obesity itself is related to higher postoperative mortality rates. Therefore, many centers may consider obesity a relative or even absolute contraindication for transplantation, depending on the body mass index (BMI) value [16].

The connection between heart failure and obesity can be evaluated from multiple perspectives. First, obesity, especially when combined with arterial hypertension, diabetes mellitus, and dyslipidemia (collectively referred to as metabolic syndrome), represents a significant risk factor for the development of heart failure and other cardiovascular diseases in general. Metabolic changes leading to HF (such as activation of the adrenergic system and RAAS pathway) are accented in obese patients. Together, they may result in left ventricular (LV) remodeling with both systolic and diastolic dysfunction [17]. A high volume of fat mass leads to an increase in circulating blood volume and stroke volume. An increment in systemic vascular resistance is also present [15]. Obesity-induced metabolic dysregulation and hyper-circulatory state further aggravate heart muscle dysfunction.

Metabolic syndrome is also associated with hyperinsulinemia and increased levels of circulating free fatty acids and adipocytokines. Diabetes mellitus also increases the risk of HF (especially HFpEF) and is associated with marked insulin resistance (IR), which is another independent risk factor for mortality in these patients. IR is also related to the severity of HF [18, 19, 20]. Despite the well-known association between obesity and cardiovascular risk factors, numerous studies have described an obesity paradox. Overweight and obese patients have lower all-cause mortality compared to regular and low-BMI patients [15, 17, 21]. This could be caused by higher metabolic reserves but also by changes in the circulating level of cytokines; adipose tissue produces soluble TNF-α receptors, which can block circulating TNF-α. This can have a cardioprotective effect. Less obese patients also have higher levels of epinephrine and renin during the examination testing [17, 22].

Notably, metabolic changes in obese patients could be, at least partially, reversible. Importance is attached to the cardiorespiratory fitness of the patient. Some studies suggest weight reduction and caloric restriction could improve the systolic and diastolic functions of the heart. Lifestyle changes (weight reduction, exercise, diet) enhance cardiorespiratory fitness and quality of life [15, 19].

### 2.2 Cachexia in Heart Failure

Several mechanisms lead to malnutrition and cachexia in chronic heart failure. Low cardiac output and fluid congestion can cause bowel swelling, malabsorption, and dyspepsia. Chronic inflammation and high circulating cytokine levels (especially TNF-α) also contribute to anorexia and lower caloric intake. Patients usually suffer from fatigue and consume a less varied diet, leading to a lack of micronutrients. Reduced energy supply, micro, and macronutrients further worsen cardiac contractile function, promote muscle weakening, and decrease exercise tolerance. Insulin resistance, often occurring in heart failure patients, impairs myocardial energetics and the anabolic effects of insulin [23]. Gut swelling can promote changes in the permeability of the intestinal wall, which can cause a translocation of Gram-negative bacteria into the bloodstream and increase TNF production. The barrier damage and lack of supplies also impair immune system function. Cachectic patients have a poor prognosis and a high readmission rate. Malnutrition is also associated with worse patient outcomes after left ventricular assist device implantation [23, 24]. Therefore, careful nutrition assessment should be conducted for every heart failure patient, including medical history and anthropometry (weight, arm circumference, triceps skin fold) [25]. An additional notable point is that BMI evaluation can be inaccurate because of edema and loss of muscle tissue.

## 3. Pharmacological Treatment of Chronic Heart Failure

The main objectives of chronic heart failure pharmacotherapy include mortality reduction, prevention of re-hospitalizations, and improvement of the patient’s overall clinical status and quality of life. As previously discussed, heart failure may be classified into three categories according to EF value (HFrEF, HFmrEF, and HFpEF), and each category has a different pathophysiological background. HFrEF is the most explored in terms of pathophysiology and treatment. Knowledge of the other heart failure types remains limited, and no therapeutic possibilities exist. Current pharmacological therapies overlap clinical characteristics and risk factors between heart failure categories. Still, it is essential to mention that evidence-based therapy only improves symptoms and prognosis in HFrEF, not in other heart failure types [1].

### 3.1 HFrEF

#### 3.1.1 RAAS Blockade

RAAS modulation remains the cornerstone of HFrEF treatment to reduce morbidity and mortality. The apparent decrease in mortality after the addition of an angiotensin-converting enzyme inhibitor (ACEi) in HFrEF has been documented repeatedly, and currently, they are recommended in all patients unless contraindicated or not tolerated [26]; they should be titrated to the maximum tolerated recommended doses as demonstrated in ATLAS trial (Assessment of Treatment with Lisinopril and Survival), which declared that high-dose regimen of lisinopril (32.5 to 35 mg daily) compared to low dose (2.5 to 5.0 mg daily) in patients with New York Heart Association (NYHA) classes II–IV and LVEF <30% leads to a significant decrease in the composite outcome of all-cause mortality plus hospitalization for HF (hazard ratio (HR) 0.85 95% CI (0.78–0.93), *p* < 0.001 for superiority). Angiotensin receptor blockers (ARBs) (the ARBs with evidence in HFrEF are candesartan, losartan, and valsartan) are indicated for NYHA classes II–IV patients with LVEF <40% who cannot tolerate ACEi or angiotensin receptor/neprilysin inhibitor (ARNI). The dose titration should be performed gradually while carefully observing blood pressure (BP), renal functions, and kalemia [26, 27].

The additional benefit of endopeptidase neprilysin inhibition in addition to RAAS blockade (angiotensin receptor-neprilysin inhibitor, ARNI) was demonstrated in the PARADIGM trial, which has proved the superiority of sacubitril/valsartan to enalapril in NYHA class II–IV patients with LVEF <40% in terms of death from cardiovascular causes (HR 0.8 95% CI (0.71–0.89) *p* < 0.001), first hospitalization for worsening HF (HR 0.79 95% CI (0.71–0.89), *p* < 0.001), and death from any cause (HR 0.84 95% CI (0.76–0.93), *p* < 0.001) [28]. As such, sacubitril/valsartan is recommended as a replacement for an ACEi in patients with HFrEF to reduce the risk of HF hospitalization and death; it also may be considered in ACEi naive (*de novo*) users based on currently growing evidence (documented in subpopulations included in PIONEER-HF and TRANSITION trial) [29, 30]. Patients commenced on ARNI should have adequate BP and an estimated glomerular filtration rate (eGFR) >30 mL/min/1.73 m^2^. When switching from an ACEi to an ARNI, the ACEi should be discontinued for at least 36 hours before starting the ARNI to reduce the risk of angioedema [26].

MRAs (mineralocorticoid receptor antagonists; spironolactone and eplerenone) have been shown to consistently improve all-cause mortality and HF hospitalization in HFrEF when added to ACEi/ARB/ARNI and beta-blockers in several trials. Thus, they are recommended in addition to standard treatment if the estimated glomerular filtration rate (eGFR) is >30 mL/min/1.73 m^2^ and serum potassium <5.0 mEq/L [26, 31, 32, 33]. The 2023 ESC guidelines update recommends finerenone as a non-steroidal selective MRA for preventing HF hospitalization in patients with chronic kidney disease (CKD) and T2DM based on the FIDELIO-DKD and FIGARO-DKD trials [2].

#### 3.1.2 Beta-blockers

The ability of beta-blockers to mitigate the neurohumoral effects in the sympathetic nervous system makes them another cornerstone of HFrEF management. Bisoprolol, metoprolol succinate, and carvedilol have been shown to reduce mortality in HFrEF significantly, and they are recommended in HFrEF unless contraindicated or not tolerated [34, 35, 36]. The ESC guidelines also include nebivolol as a treatment option based on a SENIOR trial where it was shown to be beneficial in HF patients aged ≥70 years in terms of reducing composite endpoint of all-cause mortality or cardiovascular hospital admission (HR 0.86 95% CI (0.74–0.99), *p* = 0.039). However, the decrease in all-cause mortality did not reach statistical significance [37]. Because of the lack of effect on all-cause mortality, American Heart Association/American College of Cardiology/Heart Failure Society of America (AHA/ACC/HFSA) guidelines do not include nebivolol among beta-blockers indicated for HFrEF. Beta-blockers should be initiated in clinically stable, euvolemic patients at a low dose and gradually titrated to the maximum tolerated dose with close monitoring of BP and heart rate [26, 38].

#### 3.1.3 Sodium-glucose Co-transporter Two Inhibitors (SGLT2i)

SGLT2i (dapagliflozin and empagliflozin) are recommended in HFrEF in addition to ACEi/ARNI, beta-blocker, and MRA based on DAPA-HF and EMPEROR-Reduced trials where patients with NYHA classes II–IV and LVEF <40% were included. Dapagliflozin significantly reduced the risk of hospitalization for HF (HR 0.7 95% CI (0.59–0.83)) and cardiovascular death (HR 0.82 95% CI (0.69–0.98)) in the DAPA-HF trial; the cardiovascular death risk decreased with empagliflozin in the EMPEROR-Reduced trial but did not reach statistical significance, although there was an apparent decrease in risk for composite endpoint of hospitalization for HF and cardiovascular death (HR 0.75 95% CI (0.65–0.86)). In patients receiving gliflozins, signs of hypoglycemia, genitourinary infections, fluid balance, and renal functions should be monitored periodically. Initially, a mild decline in GFR should be expected, as documented in the EMPEROR-Reduced trial (at week 4, 5.2% placebo-corrected eGFR decline from baseline was observed in CKD patients and 3.8% in those without CKD). However, at the end of the follow-up (median of 16 months), the annual rate of decline in the eGFR and serious renal outcomes were significantly lower in the empagliflozin group [26, 39, 40].

#### 3.1.4 Other Drug Classes Used in HFrEF

In patients with signs of congestion, loop diuretics are recommended at the lowest dose possible to maintain euvolemia. The addition of thiazide diuretics should be reserved for patients who do not respond to moderate- or high-dose loop diuretics to minimize electrolyte abnormalities. Ivabradine, a selective inhibitor of If channels in the sinoatrial node, has been demonstrated to be effective in terms of reducing hospital admissions for worsening HF and deaths due to HF in the SHIFT trial, and as such, it is recommended in symptomatic NYHA class II–IV patients with LVEF <35%, in SR and resting heart rate ≥70 bpm, despite treatment with maximum tolerated beta-blocker dose (or in case of intolerance/contraindication). The data regarding the benefit of digoxin in patients with HF are conflicting. According to ESC, it may be considered in symptomatic HFrEF in sinus rhythm despite optimization of GDMT while checking serum levels and maintaining a serum digoxin concentration <1.2 ng/mL [26, 41].

Among patients with NYHA classes II–IV and LVEF <45% with evidence of worsening HF despite GDMT optimization, stimulation of cyclic guanosine monophosphate (cGMP) production via soluble guanylate cyclase (sGC) is a promising direction. The VICTORIA trial demonstrated a 10% relative risk reduction in the composite endpoint of death from any cause or hospitalization from HF (HR 0.9 95% CI (0.83–0.98), *p* = 0.02) associated with the use of vericiguat as a sGC stimulator when added to GDMT. New treatment options are still warranted, especially in patients with HFrEF progression, despite GDMT optimization [42].

### 3.2 HFmrEF and HFpEF

No randomized controlled trials (RCTs) specifically address HFmrEF (LVEF 41–49%); the data are mainly derived from post hoc or subgroup analyses of trials for HFpEF or HFrEF. They have suggested the benefits of using GDMT for HFrEF (i.e., ACEi or ARBs, beta-blockers, ARNI, MRA) in terms of HFmrEF. As such, according to the latest AHA/ACC/HSA and ESC guidelines, they may be considered (class IIb) to reduce the risk of HF hospitalization and death also in the HF population with a LVEF of 41–49%. SGLT2 inhibitors were demonstrated in EMPEROR-preserved and DELIVER trials to significantly reduce the composite outcome of cardiovascular death or HF hospitalization in NYHA class II–IV patients with LVEF >40%. However, this result was driven mainly by a lower risk of HF hospitalization with no benefit on all-cause mortality; they are currently recommended in both patients with HFmrEF and HFpEF. Diuretics are also recommended in HFmrEF and HFpEF in case of congestion to alleviate signs and symptoms. As for other treatment options in HFpEF, no significant benefit on mortality associated with GDMT HFrEF has been demonstrated until now, and thus, recommended the management of HFpEF includes symptomatic relief, identification, and treatment of specific causes (i.e., amyloidosis), and treatment of comorbidities [2, 43, 44]. Lifestyle changes (weight reduction in obese patients, smoking cessation, increasing exercise) should also be considered.

### 3.3 Future Perspectives in Heart Failure Treatment

Current guidelines bring new insight into the treatment of cardiomyopathies as one of the substantial causes of HF. Currently, it is an effort to detect early, even pre-clinical forms, and provide the personalized treatment process based on phenotype evaluation and genetic testing of the disease (i.e., enzyme replacement therapy (ERT)/chaperone in lysosomal storage disease or tafamidis in wild-type amyloidosis—ATTRwt). Ongoing research trends will also likely focus on new biomarkers detection associated with HF that could improve treatment guidance. Among other directions in HF treatment that have been suggested, there are soluble guanylate cyclase stimulators, agents that loosen the bonds between proteins and glycosylation end products, and gene therapy to increase the level of sarcoendoplasmic reticulum calcium ATPase 2 (SERCA2) protein in the myocardium. Gene-editing techniques, such as clustered regularly interspaced short palindromic repeats-CRISPR associated protein 9 (CRISPR–Cas9), seem to be promising options in terms of offering a potential way to correct genetic mutations contributing to HF. In addition, tissue engineering and stem cell therapy might potentially repair damaged cardiac tissue and restore function [45, 46, 47].

### 3.4 Guideline-directed Medical Therapy in LVAD Patients

Although no prospective RCTs evaluating the efficacy and safety of HF-GDMT, specifically in the population after LVAD implantation, have been published, there is growing evidence of clear mortality benefit, especially for ACEi/ARBs in this population [48, 49]. Despite the lack of evidence regarding the efficacy of other drug classes in LVAD, according to 2023 The International Society for Heart and Lung Transplantation (ISHLT) guidelines, GDMT for HF is also reasonable to use in these patients (i.e., ACEi/ARBs/ARNI, beta-blockers, MRA, diuretics in case of congestion, SGLT2i, digoxin). The interaction between blood and device materials can induce the destruction of blood elements and the formation of thrombi. The inherent thrombogenicity can result in pump-related thrombus, potentially causing device malfunction and embolization. Therefore, anticoagulants (vitamin K agonists—VKA’s, i.e., warfarin) are necessary [50]. Antiplatelet agents such as aspirin may not be required for patients with HeartMate 3 LVADs, as their exclusion has been shown to reduce bleeding complications without increasing thromboembolic events [51].

Regarding bleeding complications, there is growing interest in using direct oral anticoagulants (DOACs) due to their predictable pharmacokinetics and the fact that there is no need for regular international normalized ratio (INR) monitoring. However, their use in LVAD patients is still under investigation, and more clinical data are needed to establish their safety and efficacy in this population. One of the promising clinical trials in this field is the DOT HM3 study [52], which investigates the use of DOACs in patients on HeartMate3 support. Initial findings from this trial suggest that DOACs offer a comparable safety profile to warfarin, with a potentially lower incidence of certain bleeding complications. However, the study also underscores the need for further extensive research and longer follow-ups to conclusively determine the efficacy and safety of DOACs in this setting [52].

## 4. Impact of Durable Left Ventricular Assist Device Implantation on Patient with Heart Failure 

### 4.1 Cellular and Metabolic Changes after LVAD Implantation

The left ventricular assist device significantly improves quality of life and survival rates in patients with advanced heart failure [15, 53]. Device therapy for end-stage heart failure may also have a favorable effect on cardiac remodeling due to hemodynamic unloading of LV [54]. Several biomarkers of myocardial fibrosis and markers of unfavorable cardiac remodeling have been identified (e.g., ST2, growth differentiation factor-15) and are diminished after LVAD support [55, 56].

It has been reported that myocardial insulin signaling improves after LVAD implantation, decreasing cardiac lipotoxicity and insulin resistance. Improvements in glycemic control (significant reduction of daily insulin dose, decline in glycated hemoglobin, and decline in fasting blood glucose level) have been described [7, 57, 58]. End-stage heart failure is accompanied by low-cardiac output (LCO) syndrome, leading to general hypoperfusion of organs. The implantation of durable LVAD increases cardiac output and improves blood circulation in affected organ systems (central nervous system, hepatic, and renal circulation) [16, 17].

Chronic heart failure can be described as a permanent inflammatory state. Despite a significant reduction in myocardial stress after LVAD implantation (volume and pressure unloading, reduction in brain natriuretic peptide (BNP)), circulating levels of inflammatory cytokines (i.e., TNF-α, IL-6, IL-8, C-reactive protein, and others) remain significantly elevated. This may result from ongoing damage to blood elements caused by the pump and systemic foreign body response [5]. Increased levels of TNF-α contribute to vascular destabilization and may play a role in LVAD-related angiodysplasia and non-surgical bleeding [59, 60]. Elevated levels of IL-6 have a pleiotropic effect on the immune system (stimulation of B-cells, increased activity of T-cells with defective responses after activation) [5]. It is important to note that the increased inflammatory response after LVAD implantation may increase the risk of various complications [61]. Mechanical unloading also leads to changes in the gene expression of sarcomeric, non-sarcomeric, and membrane-associated proteins [62]. Heart failure is associated with an altered calcium metabolic pathway (downregulated gene expression of sarcoplasmic endoreticular Ca^2+^ ATPase—SERCA; modified function of the sarcolemmal Na^+^/Ca^2+^ exchanger), which correlates with contractile dysfunction. LVAD implantation is associated with improved calcium signaling. Animal models (heterotopic heart-unloaded rats) have shown that hemodynamic unloading improves regeneration of cardiomyocytes in failing hearts by increasing cell proliferation and inhibiting cell apoptosis [62]. After left ventricular assist device implantation, there is a significant reduction in serum BNP and its cleavage fragment N-terminal pro–B-type natriuretic peptide (NT-proBNP) hormones produced by cardiomyocytes undergoing mechanical stress. Unfortunately, the mechanisms responsible for changes in myocardial metabolism in response to LV unloading are not fully understood and remain to be explained.

### 4.2 Complications and Adverse Effects after LVAD Implantation

**Fig. 2.**
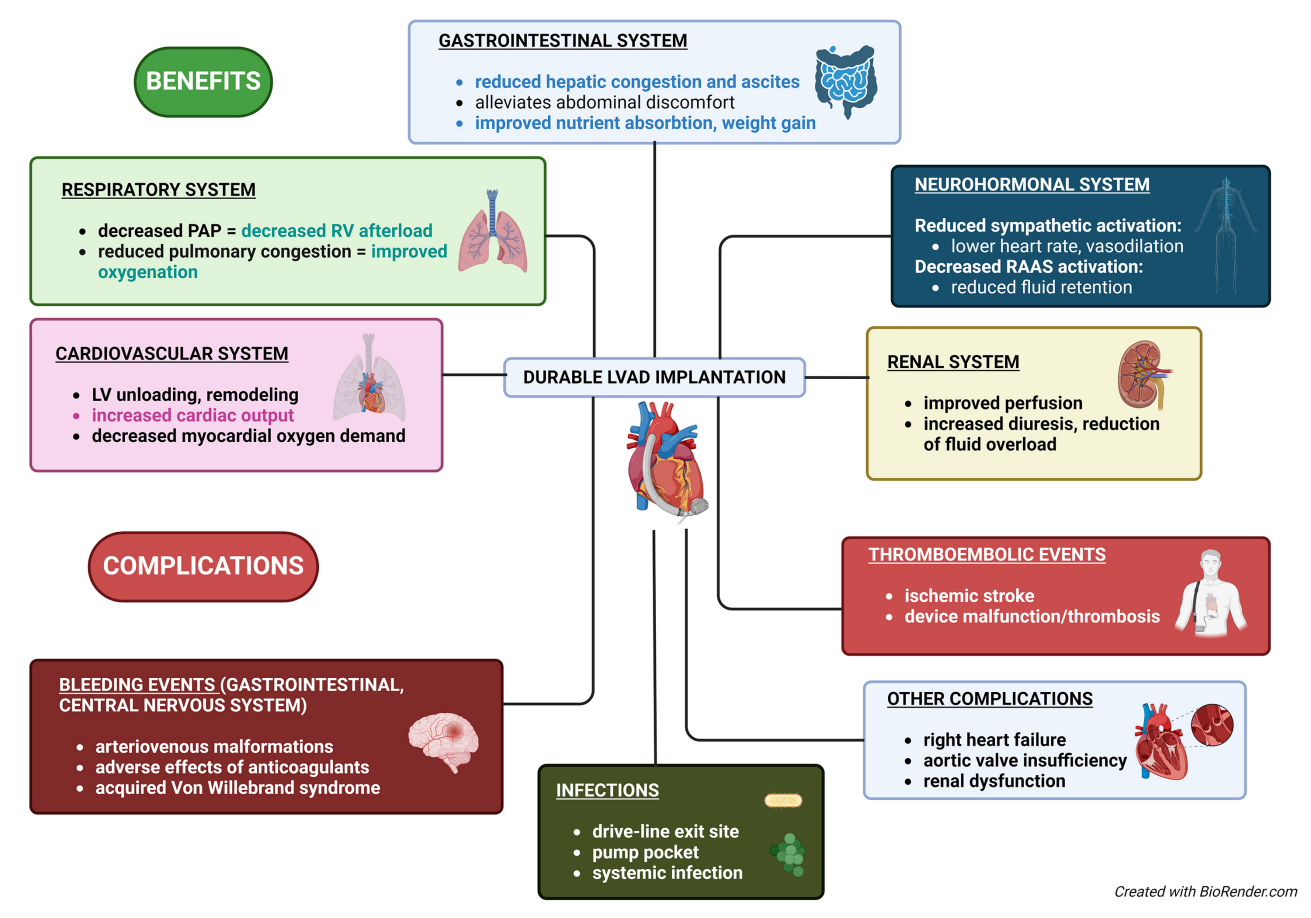
**Impact of a durable left ventricular assist device (LVAD) implantation on heart failure patients**. This diagram illustrates the multifaceted impacts of the implantation of a LVAD on heart failure patients, detailing the benefits and potential complications. PAP, a mean plulmonary arterial pressure; RV, right ventricle; LV, left ventricular; RAAS, renin–angiotensin–aldosterone system.

Recent third-generation implantable continuous-flow LVADs with advanced pump technologies (fully magnetically levitated centrifugal pump, extended durability, and thromboresistance) exhibit superior performance and significantly lower incidence of adverse events than previous generation devices [63, 64]. Several potential adverse effects of durable mechanical circulatory support (MCS) should also be considered (Fig. 2).

#### 4.2.1 Thromboembolic and Bleeding Complications

One of the most significant complications following the implantation of any LVAD is a stroke, including both ischemic and hemorrhagic. This complication may arise as an adverse effect of anticoagulation therapy, arteriovenous malformations (occurring as a side effect of LVAD continuous blood flow), or arrhythmias, such as atrial fibrillation, which increases the risk of clot formation and development of thromboembolic neurological adverse events. Given the strong association between uncontrolled blood pressure and stroke, strict blood pressure control is necessary to eliminate the risk of adverse cerebrovascular events. Cerebrovascular incidents affect 10% of patients within the first year of support and are a leading cause of death [65]. One of the factors potentially contributing to bleeding after implantation can be acquired von Willebrand syndrome (excessive cleavage of the von Willebrand factor) demonstrated in axial and centrifugal-flow pumps [66, 67]. Refinement of device technology has led to a reduction in the incidence of stroke.

Gastrointestinal (GI) bleeding occurs at unexpectedly high rates (15–40% of LVAD patients) [63, 68]. Further, patients with significant GI bleeding require an endoscopy to determine the source and provide control of any lesions; bleeding sources are predominantly from the upper GI tract [69]. The role of the difference between continuous or pulsatile pump flow remains unexplained.

Device malfunction or failure due to pump thrombosis is a rare but potentially life-threatening complication with a large variety of symptoms (ranging from no symptoms to cardiac arrest), typically necessitating an urgent pump exchange [70].

#### 4.2.2 Pulmonary Hypertension and Right Heart Failure in LVAD Patients

4.2.2.1 Pulmonary Hypertension

Pulmonary hypertension (PH) frequently occurs in patients with end-stage heart failure. The progression of PH is caused by a gradual increase in left-sided filling pressures, which leads to passive elevation of pulmonary venous and arterial pressures, resulting in vascular remodeling and increased pulmonary vascular resistance (PVR) [71, 72]. Fixed pulmonary hypertension (fPH), as a result of the vascular remodeling process, is a contraindication for heart transplantation due to the unacceptable risk for post-transplant right heart failure and elevated post-transplant mortality rate [71, 73]. Durable LVAD implantation reduces PVR by continuously unloading the left ventricle, reducing the left atrial pressure and, thus, pulmonary decongestion. Therefore, it should be considered a bridge to the candidacy strategy for patients with fPH. This approach allows hemodynamic re-evaluation of pulmonary vascular resistance and the establishment of heart transplantation (HTx) candidacy [71, 73].

4.2.2.2 Right Heart Failure

Right heart failure (RHF) is a significant complication following LVAD implantation, affecting 10–40% of patients, and is associated with a poor outcome [70, 74]. RHF may be caused by various factors, including increased cardiac output leading to a higher preload in the right ventricle (RV), a leftward shift in the interventricular septum reducing RV contraction efficiency, tachyarrhythmias, tricuspid valve regurgitation, and patient-related factors [74].

The native right ventricle has to generate adequate flow across the pulmonary circuit to fill the implanted LVAD sufficiently. Still, the capacity of the RV to handle increased preload depends on the pre-existing RV functional reserve. RHF is further worsened by hypervolemia, such as excessive blood product substitution after significant intraoperative bleeding.

There is an increased risk of RHF development in patients with pulmonary hypertension undergoing LVAD implantation, and those patients have an increased burden of postoperative morbidity and mortality, particularly in the early postoperative period. Inotropic support, diuretics, and multiple pulmonary vasodilators (e.g., inhalation nitric oxide, milrinone, sildenafil, etc.) are commonly used. However, there is a lack of supporting evidence and a need for prospective randomized controlled trials. In severe RV failure, implantation of a temporary right ventricular assist device (RVAD) is a strongly recommended life-saving option [72].

Alternatively, as mentioned above, LVAD support reduces pulmonary arterial pressures (RV afterload), leads to vascular remodeling of fixed pulmonary hypertension, reduces pulmonary vascular resistance, and improves RV function [75].

#### 4.2.3 Renal Dysfunction 

Renal dysfunction and heart failure are closely interrelated and referred to as cardiorenal syndrome (CRS). It is a bidirectional relationship in which dysfunction in one organ induces dysfunction in the other. In HF patients, reduced cardiac output, low systemic blood pressure, neurohormonal activation, and venous congestion contribute to renal dysfunction. Preoperative renal dysfunction is associated with decreased survival after LVAD implantation [76]. Renal function may be further compromised by cardiac surgery due to the use of cardiopulmonary bypass, aortic cross-clamping, blood transfusions, and vasopressors [77].

Introducing an LVAD can improve cardiac output and renal blood flow, potentially enhancing renal function. However, LVAD patients are still at risk of renal failure due to factors such as RHF, hemolysis, infection, and hypotension (i.e., due to blood loss) [76]. Regular monitoring of renal function, careful dosing of HF pharmacotherapy, and vigilant management of anticoagulation are crucial to minimizing risks of renal failure development and progression.

#### 4.2.4 Aortic Valve Regurgitation

LVAD implantation has several effects on aortic valve competence. Due to continuous high-pressure flow (supravalvular blood pressure caused by outflow cannula bloodstream), there is an altered aortic root biomechanics with a potential for remodeling and dilation. Less frequent aortic valve opening may lead to leaflet stiffness and commissural fusion. Those mentioned above may contribute to the development and progression of secondary aortic insufficiency, which is a severe complication with a poor prognosis [78]. Patients with moderate to severe aortic valve (AV) insufficiency may benefit from minimally invasive transcatheter methods such as valvular occlusion with an Amplatzer device or valvular replacement [79].

#### 4.2.5 Device-related Infections

Another serious complication resulting from the artificial surfaces of MCS pumps is infection. Device-related infections can be divided into driveline and deep wound/pocket infections. Both require aggressive anti-infectious therapy (parenteral antibiotics) and often a surgical exploration. Obese patients with mechanical circulatory support have a higher cumulative rate of infectious complications and re-operations for infection than non-obese patients [80, 81, 82, 83, 84]. Device-related infections can also contribute to coagulation abnormalities, leading to thrombus formation and increasing the risk of stroke.

## 5. Conclusions

With the increasing prevalence of heart failure and a shortage of donor organs, the number of patients who may benefit from durable mechanical circulatory support continues to grow, especially in the indication of destination therapy. Therefore, it is necessary to fully understand metabolic changes that may occur after the implantation of a durable left ventricular assist device and the impact of current heart failure pharmacotherapy (i.e., SGLT2i on body mass and arterial pressure reduction, improvement of insulin sensitivity, etc.). As mentioned above, several side effects may accompany durable mechanical circulatory support. The most devastating complications include thrombotic or hemorrhagic events. A future therapeutic target should consist of patient-tailored anticoagulation and antiplatelet therapy based on carefully evaluating patient risk factors (based on age, previous history of thrombotic or hemorrhage complications, and previous pharmacotherapy). Current MCS devices are connected to batteries using a drive-line, representing a place of less resistance to infections. Therefore, meticulous care of the driveline exit site is essential. Next-generation MCS devices may improve this issue by being fully implantable and utilizing wireless charging capabilities.

Another challenge is correctly evaluating the patient’s social, nutritional, and physical statuses and optimizing them pre/post-LVAD implantation. This may include exercise, lifestyle changes, symptom monitoring, and self-care/adherence. Finally, remotely tracking the pump parameters, weight changes, and overall patient status may also improve MCS support and the patient’s quality of life.
